# NMR-Based Metabolomic Analysis and Microbial Composition of Soil Supporting *Burkea africana* Growth

**DOI:** 10.3390/metabo10100402

**Published:** 2020-10-10

**Authors:** Lufuno Ethel Nemadodzi, Jacques Vervoort, Gerhard Prinsloo

**Affiliations:** 1 Department of Agriculture and Animal Health, University of South Africa, Science Campus, Florida, Johannesburg 1710, South Africa; prinsg@unisa.ac.za; 2 ABBERU, University of South Africa, Science Campus, Florida, Johannesburg 1710, South Africa; 3 Laboratory of Biochemistry, Wageningen University, 6708 PB Wageningen, The Netherlands; Jacques.vervoort@wur.nl

**Keywords:** soil metabolomics, *Penicillium* sp, soil microbial community, *Burkea africana*, plant growth, nuclear magnetic resonance (NMR), growth promoting metabolites (GPM)

## Abstract

*Burkea africana* is a leguminous tree used for medicinal purposes, growing in clusters, on soils impoverished from most nutrients. The study aimed to determine the factors responsible for successful reproduction and establishment of the *B. africana* trees in nature, as all efforts for commercial production has been proven unsuccessful. An investigation was carried out to determine the metabolomic profile, chemical composition, and microbial composition of the soils where *B. africana* grows (*Burkea* soil) versus the soil where it does not grow (non-*Burkea* soil). ^1^H-NMR metabolomic analysis showed different metabolites in the respective soils. Trehalose and betaine, as well as a choline-like and carnitine-like compound, were found to be in higher concentration in *Burkea* soils, whereas, acetate, lactate, and formate were concentrated in non-*Burkea* soils. Liquid Chromatography-Mass Spectrometry analysis revealed the presence of numerous amino acids such as aspartic acid and glutamine to be higher in *Burkea* soils. Since it was previously suggested that the soil microbial diversity is the major driver for establishment and survival of seedlings in nature, Deoxyribonucleic acid (DNA) was extracted and a BLAST analysis conducted for species identification. *Penicillium* species was found to be highly prevalent and discriminant between the two soils, associated with the *Burkea* soils. No differences in the bacterial composition of *Burkea* and non-*Burkea* soils were observed. The variances in fungal composition suggests that species supremacy play a role in development of *B. africana* trees and is responsible for creating a supporting environment for natural establishment and survival of seedlings.

## 1. Introduction

*Burkea africana* Hook (Wild syringa) is a medium sized leguminous tree, which belongs to the sub-family Caesalpiniaceae, usually 10–12 m in height and occasionally reaching over 20 m tall. It grows in savannas and woodlands up to 1500 m altitude and inhabits dry, acidic sandy soils impoverished in most nutrients essential for plant growth [1]. *Burkea africana* is dioecious (separate male and female trees) and produces an annual cohort of large seeds from January to July. In nutrient-poor ecosystems, seeds generally represent the largest investment a plant makes of scarce nutrient reserves [2]. This monotypic genus is dominant in Namibia, Botswana, Zambia, Nigeria, Ethiopia, and South Africa particularly in the provinces such as Limpopo, North West, Gauteng, and Mpumalanga [3]. In South Africa, it is commonly known as Mufhulu in TshiVenda, Mpulu in XiTsonga and Monato in SeTswana, with several potent medicinal properties and biological activities reported.

Traditional healers in different countries have used *B*. *africana* for diverse medicinal and health benefits. In Mali, the bark is used for the treatment of numerous ailments, comprising headache, migraine, dizziness, pain, inflammation, and thrush [4], in addition to use as an antineuralgic, wound-healing and tooth-cleaning agent [5]. Furthermore, another survey conducted in Mali revealed that traditional healers cure numerous illnesses such as malaria, gastrointestinal diseases, sexually transmitted diseases such as gonorrhea and syphilis, insects and snakebites using *B*. *africana* [4]. However, *B*. *africana* has proven difficult to propagate hence the tree is not grown commercially and consequently not found in nurseries although in very high demand by the commercial market. There is currently no information regarding soil factors, their constituents and microbes which may contribute and therefore play a huge role in the successful establishment of seedlings in nature, determining the success of growing these trees outside their natural habitat.

The field of metabolomics, studies small molecules such as amino acids, nucleic acids, lipids, or carbohydrates as well as other more complex secondary metabolites which are present in cells and/or extracellular fluids of biological organisms [6]. Metabolites are the end products of a variety of cellular processes which provide high-throughput characterization and quantification of living organisms, and as a result, are increasingly applied to the areas of system biology, drug discovery, pharmaceutical research, early disease detection, toxicology, and food science [7]. Most metabolites produced by soil microbes appear to be secreted and play a role in controlling biotic interactions. However, there is still a huge gap as not much work has been documented where metabolomics was used to address soil related dynamics and challenges thereof.

Plant interactions and feedback with soil microbes determine ecosystem functioning and primary productivity in terrestrial habitats [8,9]. Soil microorganisms and meiofauna (i.e., microfauna and mesofauna) play significant roles in nutrient cycling, by consuming living organisms as well as dead organic material, and consequently disperse these degradation products into the soil [10,11]. Fungi in soils are important, because of their role in pathogenicity, nutrient cycling, and plant nutrient uptake via mycorrhizas, yet comparatively little is known about their diversity and distribution relative to their aboveground counterparts [12]. Patterns of diversity and composition are the basic descriptors for any community. Where the natural habitat of trees comprises of poor soil conditions deprived of essential elements, it has been proposed previously that a symbiotic relationship with a fungal species in the soil, is the most probable explanation for the survival of the trees in the wild. Recent studies on natural grassland plants showed that plant species richness is positively correlated with that of several major fungal groups on a local scale [13,14,15,16]. In addition, with the advanced development of metagenomics, estimation of the fungal diversity in a variety of soil environments, such as streams phyllospheres, soils of deciduous and coniferous forests and seeds of Neotropical trees have been reported [17].

Nuclear Magnetic Resonance (NMR) spectroscopy and LC-MS analysis are commonly used to determine the metabolites profile of various samples. NMR is widely used for high-throughput characterization of metabolites in complex biological mixtures [18]. The advantage of using ^1^H-NMR has been reported as an advanced analytical method which is non-destructive and highly reproducible [19]. The number of peaks generated by a metabolite, as well as their location and ratio of heights, are reproducible and uniquely determined by the chemical structure of the molecule [18]. LC-MS is however a very accurate and fast analysis due to the use of a column, effective quantification of a broad range of known cellular metabolites, and simultaneous detection of unanticipated metabolites via untargeted analysis [20]. MS-based techniques are more sensitive, particularly when using liquid chromatography (LC) connected to a tandem MS/MS for quantitative analysis in the multiple reaction mode [21].

Metabolomics has been widely applied in several human and plant studies; however, there is still an underrepresentation of metabolomics studies on soil metabolites. The aim of the study was to investigate the variance in the chemical composition of the soils using advanced ^1^H-NMR, LC-MS, and metagenomics analysis for a comprehensive understanding of different soil metabolites and soil microbes, which contribute to the growth and establishment of *B*. *africana* trees. Extensive databases such as the human metabolome database (HMDB) [7]; BioMagResBank (BMRB) [22] and commercial databases such as the Chenomx NMR suite [23] were used to annotate NMR specific characteristics which are also known as values for a variety of metabolites. Growth promoting metabolites (GPM) such as trehalose and betaine as well as amino acids such as glutamine and aspartic acid were found to be available in higher concentrations in *Burkea* soils, and are therefore the contributing factors toward survival and resilience for *B*. *africana* trees to successfully grow amid abiotic stress, dehydration and low level of soil fertility. This study therefore provides information on the soil factors and microbial communities which are contributing to the survival of the seedlings, although more factors might also be important. These findings will play a significant role in understanding why trees do not grow outside their natural environment and this information will therefore provide assistance to tree growers to grow *B. africana* trees in nurseries and/or outside their natural habitat.

## 2. Results

### 2.1. Composition of Soils

The nitrate and ammonium levels of Burkea soils were higher (*p* < 0.05) than those found in non-Burkea soils. However, similar values (*p* > 0.05) were observed for all micro and macro minerals including total nitrogen, pH, and organic matter as presented in Table 1.

### 2.2. Annotation of Compounds

**Figure 1:**
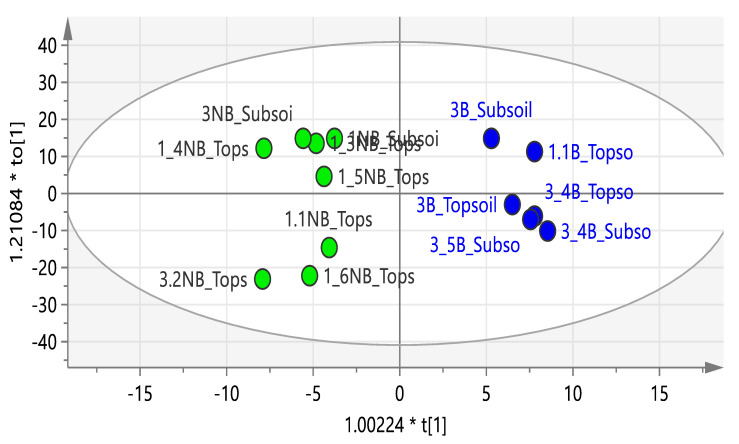
OPLS-DA used as a statistical model to show separation between Burkea (blue) versus non-Burkea (green) top and sub soils (R^2^X = 0.938 and R^2^Y = 0.593).

**Figure 2:**
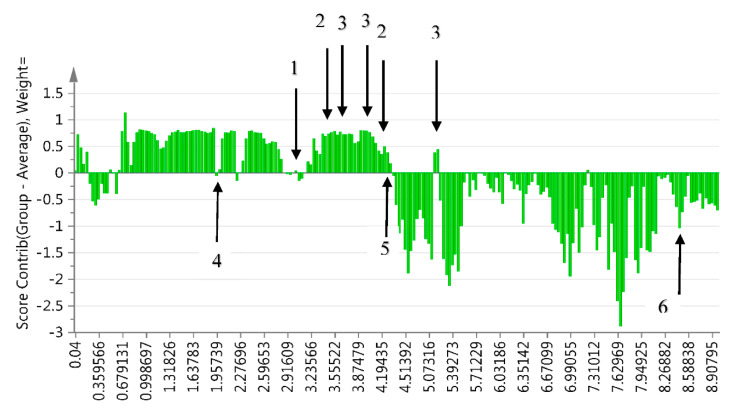
Contribution plot representing the differences between Burkea versus non-Burkea soils showing regions with positive correlation with Burkea soils for choline-like (1) (3.1 ppm), carnitine-like (1) (3.1 ppm), betaine (2) (3.3 and 3.9 ppm) and trehalose (3) (3.6, 3.8, 3.9 and 5.2 ppm) compounds and negative association for acetate (4) (1.9 ppm), lactate (5) (4.1 ppm) and formate (6) (8.4 ppm).

The difference in the metabolic profile of Burkea soils versus non-Burkea soils was detected using OPLS-DA and a contribution plot (Figure 1 and Figure 2).

Trehalose (3.6, 3.8, 3.9, 5.2 ppm), betaine (3.3 and 3.9 ppm), carnitine-like, and choline-like compounds (3.1 ppm) showed a positive correlation with the Burkea soils and a negative association with non-Burkea soils. On the contrary, acetate (1.9 ppm), lactate (4.3; 1.3 ppm) and formate (8.4 ppm) was positively associated with the non-Burkea soils as shown in Table 2. The annotation of these metabolites was done using Chenomx Profiler as prescribed by [6], Human Metabolome Database and previously published data.

### 2.3. Identification of Annotated Metabolites

Trehalose and betaine were positively identified by spiking the samples, whereas choline and carnitine were almost identical to the observed chemical shifts of the standards on the NMR analysis. It is due to the fact that reason that these compounds in Burkea soils were therefore labeled as choline-like and carnitine-like.

The metabolic profile of Burkea soils and non-Burkea soils analyzed using the LC-MS are shown in Table 3. Higher (*p* < 0.05) concentrations of aspartic acid, serine, 4-hydroxyproline, glutamine, threonine, glutamic acid, citrulline, proline, lysine, guanosine, tyrosine, adenosine, isoleucine, phenylalanine, and tryptophan were prevalent in Burkea soils than in non-Burkea soils. However, the concentration levels of glycine, cytidine, adenine, leucine, acetylcarnitine and fumaric acid were similar (*p* > 0.05) in both Burkea soils and non-Burkea soils.

### 2.4. Bacterial Community Composition

*Burkea* and non-*Burkea* soils did not show great difference in the bacterial composition. Both soils possessed the same taxonomical kingdoms comprising of bacteria (99%), archaea (0.05%), protozoa (0.01%) with a very small amount of an unknown taxonomical kingdom. Furthermore, the results also revealed that the main dominant phylum that ranked first consisted of unknown, unidentified, and uncultured bacteria (99.03%), which could not be grouped with the sequence of known bacteria phylum classification. The second leading phylum in *Burkea* soils was Proteobacteria (3.52%), which was distributed as Alphaproteobacteria (0.10%)*,* Betaproteobacteria (0.02%), Deltaproteobacteria (0.01%) and (0.01%*)* Gammaproteobacteria. The Actinobacteria (1.33%), was ranked third, followed by Acidobacteria (0.76%), Planctomycetes (0.20%), Firmicutes (0.14%) and Verrucomicrobia (0.01%).

#### Order and Family and Species Classification

In total 92.20% comprised of unknown and unidentified bacterial class with Plactomycetes and Alphaproteo bacteria occupying 3.59–2.21% respectively. A small percentage (0.54–0.16%) was made up of Acidobacteria, Chloroflexi, Gymnostomatea, Caldilineae and Betaproteobacteria. A larger proportion of unknown order of bacteria showed a high occupancy of 93.03%. However, a small distinct noticeable order of Planctomycetales was prevalent in non-*Burkea* soil although in small quantities comprising only 3.59%. In addition, Rhizobiales occupied only 0.84%. Furthermore, Rubrobacterales, Bacillales and Acidobacteriales occupied the lowest percentage ranging from 0.42–0.07% respectively. BLAST output results in both *Burkea* and non-*Burkea* soil showed the same level and percentage of uncultured bacteria and 16 RNA at 45.65% each. Uncultured Firmicutes occupied 6.72% while uncultured bacteria occupied 3.37%. The main and only bacterial difference between *Burkea* soils and non-*Burkea* soils was the presence of Chloroflexi phylum, which formed part of the phylum classification in non-*Burkea* soils.

### 2.5. Taxonomical Kingdom and Phylum Classification of Fungal Composition

*Burkea* soils: The results found in *Burkea* soils showed that kingdom classification was assigned as plantae (63.83%), fungi (29.75%), protozoa (4.06%), bacteria (0.99%) with 1.35% reading counts which remained unknown. In addition, phylum classification had diverse soil communities with Tracheophyta identified as the dominant phylum comprising of 63.82% in *Burkea* soils. The second prevalent was Ascomycota which is a fungal phylum with 16.63%, followed by Ciliophora with 4.06%.

Non-*Burkea* soils: Kingdom classification in non-*Burkea* soils was dominated by fungi (73.15%), followed by bacteria (10.09%), protozoa (9.78%), unknown (5.52%) and lastly plantae (1.45%). Phylum classification showed a variety of soil microorganisms representing the different kingdoms mentioned above. Ascomycota (44.15%) proved to dominate, followed by an unknown fungal phylum (36.36%), Ciliophora (9.78%), Basidiomycota (7.63%), Tracheophyta (1.36%), Glomeromycota (0.43%) and Actinobacteria (0.38%). Other phyla found in these soils with very low concentrations were Bryophyta, Zygomycota, Chytridiomycota and Bacteriodetes (0.48%).

#### 2.5.1. Order and Family Classification

Asparagales (63.81%) dominated *Burkea* soils followed by an unknown fungal species at 16.82%. Coniochaetales and Hypocreales showed percentages of 7.74% and 7.89%, respectively. Furthermore, Spathiididae also formed part of the top order classification with 4.06%. *Orchidaceae* (51.90%) dominated *Burkea* soils, followed by 33.29% of unknown families. *Spathidiidae* (10.49%) was ranked the third, followed by *Trichocomaceae*, *Coniochaetaceae* and *Bionectriaceae* (0.49%). Lower percentages were occupied by *Hyaloscyphaceae, Netriaceae* and *Amphisphaeriaceae* (0.29%).

On the contrary, non-*Burkea* soils showed that *Eurotiales* took dominance with 84.52%, unknown and unidentified families occupied 48.55%, followed by *Trichocomaceae* and *Sphathidiidae* (25.03%).

#### 2.5.2. Identification of Fungal Species

**Figure 3:**
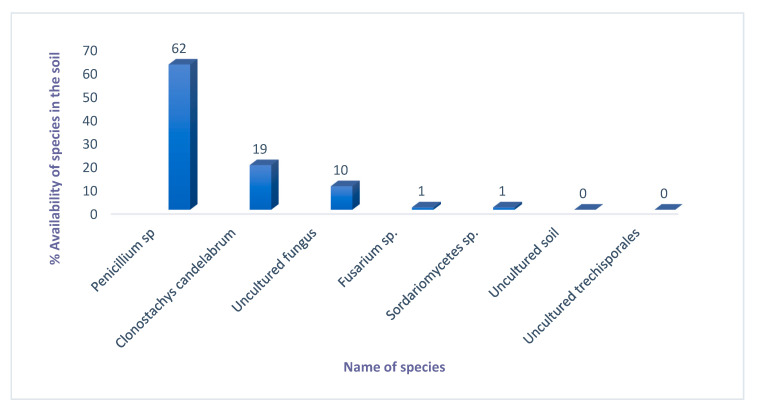
Representation of fungal species in *Burkea* soils.

*Penicillium* sp. highly dominated *Burkea* soils with 72.17%, followed by *Clonostachys candelabrum* (22.53%) and an uncultured fungal species (Figure 3).

**Figure 4:**
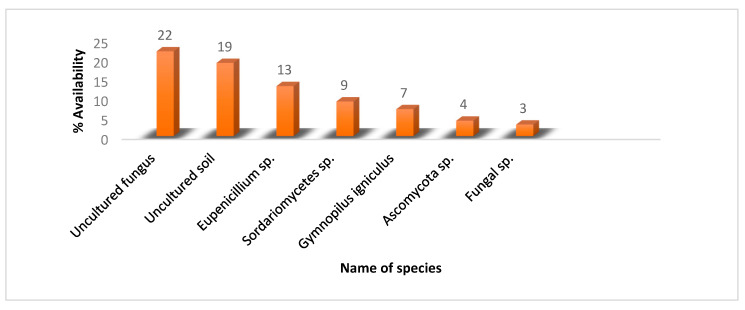
Representation of fungal species in non-*Burkea* soils.

Non-*Burkea* soil was dominated by uncultured fungi and uncultured soils, which could not be categorized nor identified under any fungal species as shown in Figure 4.

## 3. Discussion

Soil is a habitat for a vast, complex, and interactive community of naturally occurring soil organisms, whose activities largely determine the physico-chemical properties of the soil. Moreover, soil microbes perform important functions in agroecosystems including their role in plant growth promotion through mineral nutrition and control of phytopathogenic microbes. From seed germination until a plant reaches maturity, it lives in close association with soil organisms [24]. Over the years, *B. africana* trees have proven difficult to grow outside their natural habitat and transplanting them have only been successful for a period of 6–8 months, which is ultimately followed by death. The results of this study proposed that specific soil metabolites play an important role in the survival of these trees, with the aid from the microbial composition which assists in promoting growth in their natural habitat. Previously, no research has been conducted on the soils surrounding *B*. *africana* trees, their nutrients status, metabolomic profile, amino acids presence and microbial communities, which probably explain the absence of these trees in the nurseries and the reasons they have not been grown successfully commercially.

Nutrient analysis showed no significant differences between *Burkea* soil and non-*Burkea* soils, with the exception of ammonium and nitrate which were predominately higher in the *Burkea* soils. Several researchers revealed that the relative amounts of ammonium and nitrate may induce, and be critical for, growth and morphogenesis of plant cells [25]. When ammonium and nitrate are both available in the soil, immobilization depletes first or exclusively the ammonium pool and nitrate only immobilized after ammonium has been exhausted. Consequently, nitrate is potentially more available in soil, particularly for plant uptake although ammonium has been found to be the preferred form of nitrogen for assimilation by microbes in many cultivated soils [26,27,28]. Generally, most crop plants prefer a mixture of ammonium and nitrate and will take up a higher proportion of ammonium to nitrate. The results of the study revealed that *Burkea* soil contains higher concentrations of ammonium and nitrate as a source of nitrogen for effective growth and establishment, especially since they grow in nutrient-poor soils.

^1^H-NMR metabolomic analysis clearly separated the *Burkea* soils from the non-*Burkea* soils into two clusters, indicating a distinct chemical profile for the two soils. A contribution plot was used to identify the NMR regions, responsible for clustering of the soil samples. The different metabolites detected with the use of ^1^H-NMR in *Burkea* soils versus non-*Burkea* soils are assumed to represent composition influenced by different microbes present and their symbiotic activities, which ultimately influence growth or on the contrary inhibit it. Trehalose, betaine, choline-like and carnitine-like compounds were found to be highly dominant in *Burkea* soils. It is proposed that trehalose, choline and betaine are growth promoting metabolites (GMP) required to sustain the growth of *B*. *africana*. These compounds are therefore present as a result of the microbial community differentiation between the *Burkea* and non-*Burkea* soils, and highlight the interaction of the microbial communities to support the growth of the trees. There is a general agreement that the production of these compounds can protect plants from stress, even when they are present at low and osmotically insignificant levels [29]. The absence of these GMP in non-*Burkea* soils is a clear indication that different soils possess different primary and secondary metabolites, which are likely to contribute to the growth of plants.

Trehalose is a peculiar non-reducing disaccharide with the units linked through an ∝,∝-1,1-glycosidic linkage [30]. Although it is known to be present in a wide variety of organisms such as yeast, fungi, bacteria, insects, some invertebrates, and lower and higher plants, it seems to perform many roles depending on the host. Expanded research work is however still undergoing to unearth and comprehend its specific, exact, and main roles. In the 1970s, trehalose was merely regarded as a storage form of glucose for energy and/or for cellular components structure [31]. In yeast and plants, trehalose has been proven to serve as a signaling compound which is able to regulate metabolic pathways [32]. In addition, trehalose act as a chemical chaperone [33] by stabilizing proteins in their native structure and thereby preventing cellular damage from inactivation or denaturation caused by stress conditions such as desiccation, dehydration, heat, cold, and damage by oxygen radicals [30]. It is also known to play a protective role during abiotic stress as an energy source and protects some plants from several pathogens [34]. For instance, in tobacco (*Nicotiana tabacum*), trehalose have improved growth under drought stress [35], and in common bean (*Phaseolus vulgaris*) nodules, trehalose accumulation is correlated with whole-plant drought tolerance. A study conducted on *Escherichia coli* revealed that increased production of trehalose resulted in increased growth under osmotic stress [36]. Several studies revealed that in root and nodule systems where available water decreases, stress causes an increase in trehalose levels in nodules. A study conducted on rhizobia supports the microbial interaction of fungal communities with leguminous plants, where it was revealed that trehalose is a key compound for signaling plant growth, yield and adaptation to abiotic stress, and its manipulation has a major agronomical impact on growth and development in leguminous plants [37,38].

Betaine has also been reported to improve growth and yield of water-stressed tobacco [39], *Zea mays* [40], and *Bacillus subtilis* [41]. Naturally occurring betaines have been reported to serve as organic osmolytes, for protection against osmotic stress, drought, salinity, or high temperature as it is responsible for water retention in cells, thus protecting from the effects of dehydration [42]. Choline was reported to serve as a nitrogen source and growth stimulant as application of choline with inorganic N improved growth in *B. rapa*. It also enhances plant growth by stimulating the photosynthetic activity in protoplasts, and foliar application enhances the growth of grass species such as Manila grass (*Zoysiamatrella Merr*.) and bent grass (*Agrostis stronifera*) [43,44]. The presence of trehalose, betaine and a choline-like compounds found in *Burkea* soils associated with microbial communities in the soils, therefore supports growth and survival of the seedlings at various stages of growth and development.

The presence of acetate, formate and lactate in non-*Burkea* soils, are linked to bacterial metabolism as they are known to be produced by bacteria [45], and probably evident of the bacterial dominance in non-*Burkea* soils. In addition, lactate was found to be an important end product of bacterial fermentation of glucose and other carbohydrates [46]. Complete growth inhibition by formate has been reported for *Thiobacillus neapolitanus, Thiobacillus thioxidans* and *T*. *ferrooxidans* [47]. Furthermore, it has been reported that high concentrations of formate could reduce the pH gradient, and as a result, inhibit the growth of cells [48].

The LC-MS results indicated that a total of 22 metabolites were identified in both *Burkea* and non-*Burkea* soils, although in different concentrations as shown in Table 3. Most of these metabolites are known to serve as energy sources for soil microorganisms and as important sources of N for plants [49]. For instance, it is reported that glutamine can serve as an alternative nitrogen source; however, high concentrations of glutamine can also inhibit growth [50]. It is, therefore, concluded that glutamine serve the same purpose as ammonia and nitrate, which were found to be significantly predominant in *Burkea* soil. Glutamine can serve as a readily available source of nitrogen as it has been reported that the release of ammonium is due to the hydrolysis of amides such as asparagine and glutamine residues in soil organic matter [51]. Based on the above findings, it is proposed that the compounds which are highly dominant in *Burkea* soils serve as growth promoters, stress protectants and as nutrient sources, especially N to compensate for the nutrient-poor soils where *B*. *africana* grows.

The microbial biodiversity conducted between *Burkea* and non-*Burkea* soils showed similarity in the bacterial community profile found in both soils. The likeness appears at the phylum level, where 94.03% of bacterial community found were unknown and seem to be in abundance. Therefore, the findings of the current study suggest that soil bacteria present in *Burkea* and non-*Burkea* soils are not plant growth promoting rhizobacteria (PGPR), which do not promote and influence growth; showing no symbiotic relationship with the roots of *B. africana* trees. The results of this study are in agreement with other studies [52,53] which revealed that soil microbial diversity is vast, and it is estimated that 99% of species remain unidentified.

Investigation into the fungal composition of *Burkea* and non-*Burkea* soils revealed that Tracheophyta (referring to plant remains) was the main phylum, followed by Ascomycota. Species classification and identification clearly quantified the differences in microbial or eukaryotic community composition between *Burkea* and non-*Burkea* soils respectively. From *Burkea* soils, a dominant *Penicillium* sp. was identified by means of BLAST analysis from the soil DNA. *Penicillium* is a well-known and most common fungi occurring in a diverse range of habitats, from soil to vegetation to air, indoor environments, and various food products [54]. Its main function in nature is the decomposition of organic materials, where species cause devastating decompositions as pre-and postharvest pathogens on food crops [55,56,57] as well as producing a diverse range of mycotoxins [58]. Its biggest impact is the production of penicillin (antibiotic), which revolutionized medical approaches to treating a wide range of bacterial infections and diseases [59,60,61,62].

Fungal species provide different benefits to their hosts [63], and more diverse communities are more efficient in the uptake of organic phosphorus [64]. Fungal species have demonstrated to respond to different plant primary and secondary metabolites that may function as carbon substrates and/or growth modifying signals [65]. Root exudates may serve as a selective agent through which a plant is able to regulate the fungal community in the surrounding rhizosphere. In addition, fungi supply inorganic nutrients to plants, such as ammonium, nitrate, and phosphate [66], and they are used as biofertilizers. Furthermore, fungi is known to produce a wide range of bioactive metabolites, which can improve plant growth [67]. Several reports have suggested that *Penicillium* sp. interact with the roots of crop plants to enhance plant growth [68,69,70]. In addition, *Pencillium* sp. is also known as a potent plant growth promoting fungi, which secrete the plant hormones, indole-3-acetic acid (IAA) and GA, and is also involved in phosphate solubilization, which may be a reason it increase the plant growth [71,72]. Furthermore, some species of *Penicillium* are well-known for their antagonistic activity against pathogens by producing antibiotics and induce resistance in plants by activating multiple defense signals [73]. In addition to the above functions, *Penicillium* can survive under environmental stress conditions such as saline soil and promote plant growth against salt stress [74]. Recently, [75] reported that *Penicillium* EU0013 inoculation is capable of enhancing growth and protecting tomato plants against *Fusarium* wilt. The results of this study suggest that there is a positive synergistic relationship between fungi in the soil and the roots of *B*. *africana* trees, which promote and influence their growth from seedling stage to maturity. Furthermore, the results suggest a strong relationship between microbial communities (fungi) and the metabolites required for the effective growth of *B. africana* trees. Another study [70], agrees with the findings of the current study, when they discovered that fungi produce a wide range of bioactive metabolites, which can improve plant growth. Therefore, excavating and/or growing *B*. *africana* seedlings from their natural habitat may distort their interaction with plant growth promoting fungal species, which may cause severe stress conditions, and ultimately death of these trees due to the absence of *Penicillium* sp., and related beneficial effects of the fungal species in the new soils.

It is, therefore, concluded that the presence and composition of soil microbes (fungi) in *Burkea* soils is important for producing GPM which may play an important role in protecting plants from adverse stress conditions or create positive and favorable conditions for establishment and growth of *B*. *africana* seedlings. The higher concentration of metabolites such as betaine, trehalose, and glutamine, therefore indicate the importance of these compounds in mitigating stress and to supply necessary nutrients to the seedlings until they reach maturity and pod bearing stage. The results of this study suggest that it could be due to the specific metabolites composition in the soils that ensures and promotes growth and survival of these trees hence *B. africana* trees are unable to grow outside their natural habitat, and most probably explain why cultivation of the trees were unsuccessful to date.

However, future research is highly recommended to determine the metabolic pathways and networks, which will show the relationships and the link(s) between the growth promoting metabolites and soil microbial composition on their role in growth thereof. In addition, inoculation of *Penicillium* sp. into non-*Burkea* soils as a growth promoting fungi for successful growth and establishment of *B. africana* trees should be further investigated.

## 4. Materials and Methods

### 4.1. Sampling Site

The study was conducted at Telperion Game Reserve, which is situated in Mpumalanga, South Africa. Three different sites within the reserve namely site 1 (25°42′40.00″ S; 029°00′21.6″ E); site 2 (25°41′26.6″ S; 029°01′46.7″ E) and site 3 (25°39′49.4″ S; 029°01′59.7″ E) were used. The reserve is approximately 1000 ha in size and is comprised of vegetation cover which is described as highveld grassland and savannah with large rocky outcrops present throughout the area [76]. It was also observed during the study that the area is dominated by sandy soils, large rocks and characterized by different species of grasses, shrubs, variety of trees and diversity of wild animals.

### 4.2. Soil Collection

Soil samples were collected from three different sites in the Telperion Nature Reserve, with two sampling regions for each site representing three areas where *B. africana* grows (*Burkea* soils) as well as 3 areas where *B. africana* does not grow (non-*Burkea* soils). The *Burkea* soils represented the rhizosphere and the non-*Burkea* soils represented the non-rhizosphere soils. However, as the trees grow in sandy soil and movement of nutrients and compounds are expected, the *Burkea* soil consisted mainly of the rhizosphere, but also the immediate soil surrounding the roots. The *Burkea* soil therefore comprised of soil collected up to 2 cm from the tree roots. The soil surface was cleaned from any plants debris and fallen leaves were removed before collection. At each site 15 soil samples were collected, which comprised of topsoil (0–30 cm) and subsoil (30–60 cm). The samples were placed in brown bags, and placed in a cooler bag, transported to the laboratory where it was stored in an ultralow freezer at −80 °C to prevent alteration of nutrients and proliferation of the microbial community until use.

### 4.3. Soil Nutrient Analysis

From the three sampling sites, 15 soil samples were randomly collected where *B. africana* trees grows, referred to as *Burkea* soils and where *B. africana* trees does not grow, referred to as non-*Burkea* soils. From these samples, three combined samples were collected and these three replicates submitted for soil analysis. Nutrient analysis of the soils was conducted at Agricultural Research Council-Soil Climate and Water (ARC-SCW) for total nitrogen, phosphorus, organic matter, pH, potassium, iron, magnesium, manganese, calcium, sodium, nitrate, and ammonium. Exchangeable cations and anions were extracted with 1 M ammonium acetate (1:10, soil: extractant ratio), shaken for 2 h and analyzed for C, Ca, Mg, K, Fe, total N and Na using automatic absorption spectrophotometry (Pharmacia LKB-Ultrospec III, Pharmacia LKB Biotechnology, Uppsala, Sweden). Exchangeable anions were extracted with distilled water (1:5, soil: H_2_O), shaken for 2 h and NO_3_^−^ and NH_4_^+^ ions in extracts were subsequently analyzed by ion chromatography (Dionex DX 120, Thermo Scientific, Johannesburg, South Africa). The pH was determined with a pH meter (Micro pH 2001, Crison, Algete, Spain in a 1:10 *w*/*v* suspension of 5 g of each sample. Organic matter content was estimated from the determination of carbon using the combustion method with the elemental analyzer (Euro EA, Eurovector, Milan, Italy).

### 4.4. Statistical Analysis

Variation in nutritional composition data for *Burkea* and non-*Burkea* soils was analyzed using a one-way analysis of variance with SAS statistical analysis software (SAS, 2010, SAS Institute, Cary, NC, USA). Mean separation was done using least significant difference (LSD) at a 5% significance level.

### 4.5. NMR Metabolomics Analysis

For NMR analysis, deuterated methanol (CD_3_OD), KH_2_PO_4_, sodium deuterium oxide (NaOD), trimethylsilyl propionic acid sodium salt (TSP) and deuterium oxide (D_2_O) was supplied by Sigma-Aldrich (Darmstadt, Germany. The buffer was prepared by adding 1.232 g KH_2_PO_4_ to 100 mL of D_2_O with 10 mg TSP (0.1%) added as a reference standard. The pH of the solution was adjusted to pH = 6.

The protocol designed by [77] was implemented for the extraction procedures, with a few adjustments. A 500 mg soil sample was transferred to 2 mL Eppendorf tubes and extracted with 750 µL deuterated methanol and 750 µL KH_2_PO_4_ buffer in D_2_O (pH 6.0) containing 0.1% TSP. The Eppendorf tubes were vortexed for 1 min at room temperature and ultra-sonicated for 20 min without heating. The solutions were centrifuged for another 15 min at 10,000 rpm to separate the supernatant from the precipitate. The supernatant was transferred to standard 5 mm NMR tubes and subjected to ^1^H-NMR analysis.

The ^1^H-NMR measurements were performed on a Varian 600 MHz spectrometer (Varian Inc., Palo Alto, CA, USA with a frequency of 599.74 MHz. The acquisition time of each ^1^H-NMR spectrum was 7 min, which consisted of 32 scans with a width of 20 ppm. Gradient shimming was used to improve homogeneity on the magnetic field. All spectra were phase corrected and binned at 0.04 ppm using MestReNova [78] before statistically analyzed with SIMCA 13.0.3 (Umetrics, Umea, Sweden). The data was scaled using the Pareto method and an unsupervised Principal Component Analysis (PCA) and a supervised Orthogonal Partial Least Squares Discriminant Analysis (OPLS-DA) model was used to illustrate the distinctive separation between the three sampling sites where soils were collected [79,80].

Metabolites identification and quantification were carried out using Chenomx NMR suite (8.6, Edmonton, AB, Canada), Metabolites Reference Libraries and the built-in spectral library of metabolites. Additional Metabolite Reference Libraries such as the Human Metabolome Database (HMDB.ca) [42] was used to confirm annotation of compounds.

### 4.6. Identification of Compounds

Annotation based on comparison to an authentic standard as prescribed by [80] was used as the criteria considered in the annotation and identification of metabolites. Standards of trehalose, betaine, choline and carnitine were individually added to the soil samples for identification of the annotated compounds. Positive identification were made with a match of all the NMR peaks of the standard in the sample.

### 4.7. LC-MS Analysis

Soil samples (10 µL injection) were analyzed in triplicate on an Agilent Infinity 1290 LC equipped with a Waters BEH C18 column (2.1 mm × 150 mm, 130°A, 1.7 m particle size) coupled to an Agilent 6490 Triple Quadrupole system with Funnel Technology (Agilent Technologies, Santa Clara, CA, USA). For quantification of metabolites, targeted, standardized and quality controlled metabolic phenotyping was performed based on LC-QQQ-MS; LCMS-8040) analysis using the PFPP method as described by [81,82,83,84]. Preparation of soil samples were done in the same manner as for ^1^H-NMR described above. The sample injection volume was 1 µL, with a single analysis of 25 min.

### 4.8. Soil Genome and Microbial Community Analysis

Soil samples (500 mg) were subjected to DNA extraction using a NucleoSpin Soil DNA kit (Mo Bio Laboratories) according to the manufacturer’s instructions. Briefly, the DNA of the soil was quantified using a Nanodrop spectrophotometer (Nanodrop Technologies, Wilmington, DE, USA) and results confirmed with agarose gel before sending for Polymerase Chain Reaction (amplification and cloning of DNA) and sequencing at Inqaba Biotechnology industry, Pretoria, South Africa.

16S rRNA: regions were amplified in a 25 µL reaction tube using Q5^®^ Hot start High-Fidelity 2x Master Mix (New England Biolabs, Country Road, Ipswich, MA, USA). An Amplicon library PCR was separately performed in replicate extractions. The DNA primers used was Truseq Tailed 341F and 785R. The Thermocycler settings for PCR amplification were as follows: (1) initial denaturation at 95°C for 2 min (2) 30 cycles of 95 °C for 20 s (3) 55 °C for 30 s (4) 72 °C for 30 s and final elongation at 72 °C for 5 min. The PCR products were purified using a Zymoclean gel DNA recovery kit (Zymo Research, Orange, CA, USA). Purified amplicons were barcoded using the NEBnext Multiplex oligos for illumina indices. The indexed amplicon libraries were purified using the Agencourt^®^ Ampure^®^ XP bead protocol (Beckman Coulter, Lakeview Parkway S Drive, Indianapolis, IN, USA). Library concentration was measured using Nebnext Library quant kit (New England Biolabs, Ipswich, MA, USA) and quality validated using Agilent 2100 Bioanalyser (Agilent Technologies, Santa Clara, CA, USA). The samples were pooled in equimolar concentrations and diluted to 4nM based on library concentrations and calculated amplicon sizes. The library pool was sequenced on a MiSeqTM (Illumina, San Diego, CA, USA) using a MiSeqTM Reagent kit V3 600 cycles PE (Illumina, San Diego, CA, USA). The final pooled library was at 10 pM with 20% PhiX as control. Data of 2 × 300 bp long reads per sample were produced. The list of primers used for bacterial sequencing were: Truseq 341 FTGACTGGAGTTCAGACGTGTGCTCTTCCGATCTCCTACGGGNGGCWGCAG and Truseq 785R ACACTCTTTCCCCACACGACGCTCTTCCGATCT GACTACHVGGGTATCTAATCC.

The list of primers used for fungal sequence were Truseq ITS1 FTGACTGGAGTTCAGACGTGTGCTCTTCCGATCTCTTGGTCATTTAGAGGAAGTAA and Truseq ITS4 ACACTCTTTCCCCACACGACGCTCTTCCGATCTTCCTCCGCTTATTGATATGC. On the High-throughput sequence, BLAST was used to indicate the relatedness or differences of microbial diversity found in *Burkea* as compared to non-*Burkea* soils, using 16S rRNA gene sequencing for a comprehensive understanding of the soil DNAs.

## Figures and Tables

**Table 1:** Nutrient composition of Burkea versus non-Burkea soils.

Soils			
Nutrient (mg/kg)	Burkea	Non-Burkea	SEM
Fe	30.26	20.27	6.070
Mn	27.38	24.76	5.964
pH	4.72	4.81	0.047
P	5.82	7.04	2.844
C	52.86	55.05	8.536
Mg	35.89	23.4	6.081
K	64.65	74.74	7.830
Na	1.78	2.56	0.917
Total nitrogen	0.13	0.16	0.001
N-NO_3_^−^	7.06 ^a^	5.35 ^b^	2.423
N-NH_4_^+^	9.40 ^a^	7.11 ^b^	0.912
Organic matter	3.40	2.57	0.964

^a,b^ Means in the same row with different superscripts are significantly different (*p* < 0.05). SEM: standard error of mean.

**Table 2:** Annotated compounds showing NMR regions found in Burkea soils versus non-Burkea top and subsoils compared to Chenomx and Human Metabolome database values.

Metabolite	NMR Region (ppm)	Chenomx	HMDB
***Burkea* Soil**			
Trehalose	3.46	3.6	3.42
	3.65	3.8	3.44
	3.83	3.9	3.65
	3.86	5.2	3.83
	3.88		3.87
	5.2		5.18
Betaine	3.27	3.3	3.25
	3.9	3.9	3.89
Choline-like	3.10	3.1	3.1
Carnitine-like	3.1	3.1	3.1
**Non-*Burkea* Soil**			
Acetate	1.92	1.9	1.91
Formate	8.46	8.4	8.44
Lactate	4.1	1.3 4.1	1.3 4.1

**Table 3:** Metabolites concentrations detected in Burkea and non-Burkea soils using LC-MS quantification.

Metabolites	*Burkea*	Non-*Burkea*	SEM
Aspartic acid	54,572 ^a^	16,087 ^b^	1739.845
Serine	366,826 ^a^	170,977 ^b^	14,905.66
4-Hydroxyproline	10,540 ^a^	7643 ^b^	447.987
Glycine	54,636	50,491	6662.825
Glutamine	130,176 ^a^	60,259 ^b^	6662.825
Threonine	66,867 ^a^	16,025 ^b^	1224.934
Glutamic acid	82,005 ^a^	13,149 ^b^	4022.601
Citrulline	22,565 ^a^	3089 ^b^	947.719
Proline	128,680 ^a^	26,096 ^b^	4530.320
Lysine	49075 ^a^	14,903 ^b^	2984.130
Guanosine	81,611 ^a^	36,711 ^b^	2731.467
Cytidine	35,296	34,131	1692.545
Adenine	134,064	125,639	19,526.543
Tyrosine	33,072 ^a^	16,720 ^b^	1379.953
Adenosine	768,010 ^a^	547,474 ^b^	18,855.132
Isoleucine	322,050 ^a^	72,972 ^b^	6094.5899
Leucine	282,818	257,661	7148.160
Phenylalanine	137,183 ^a^	35,000 ^b^	7738.684
Acetylcarnitine	75,057	63,587	366.627
Tryptophan	41,518 ^a^	15,248 ^b^	1480.327
Fumaric acid	336,355	335,903	4209.594

^a,b^ Means in the same row with different superscripts are significantly different (*p* < 0.05). SEM: standard error of mean. *The intensities reported are relative intensities.*

## Data Availability

The data for the study was deposited in a publicly available database: G.P.; L.E.N.; J.V. (2020), “Burkea africana”, Mendeley Data, V1, doi: 10.17632/59c4853by5.1 http://dx.doi.org/10.17632/59c4853by5.1. The data can be accessed at: “NMR metabolomics” DOI: http://dx.doi.org/10.17632/59c4853by5.1#folder-a7e43083-16b7-483f-a655-32bbfd20f3bd; “Soil analysis” DOI: http://dx.doi.org/10.17632/59c4853by5.1#folder-5c522102-44f5-4c69-aa01-84b2f1377b0f; “Metagonomics” DOI: http://dx.doi.org/10.17632/59c4853by5.1#folder-fac40248-ea01-4f49-bf4f-c47eb63c2a48.

## References

[1] Marschner H. (1995). Mineral Nutrition of Higher Plants.

[2] Witkowski E.T.F., Lamont B.B. (1996). Disproportionate allocation of mineral nutrients and carbon between vegetative and reproductive structures in Banksia hookeriana. Oecologia.

[3] Wilson B.G., Witkowski E.T.F. (2003). Seed banks, bark thickness and change in age and size structure (1978–1999) of the African savannah tree, Burkea africana. Plant Ecol..

[4] Malterud K.E. (2017). Ethnopharmacology, Chemistry and Biological Properties of Four Malian Medicinal Plants. Plants.

[5] Mathisen E., Diallo D., Malterud K.E. (2002). Antioxidants from the bark of Burkea africana, an African medicinal plant. Phytother. Res..

[6] Wishart D.S., Tzur D., Knox C., Eisner R., Guo A.C., Young N., Cheng D., Jewell K., Arndt D., Sawhney S. (2007). HMDB: The Human Metabolome Database. Nucleic Acids Res..

[7] Gowda G.A.N., Zhang S., Gu H., Asiago V., Shanaiah N., Raftery D. (2008). Metabolomics-based methods for early disease diagnostics. Expert Rev. Mol. Diagn..

[8] Wardle D.A., Bardgett R.D., Klironomos J.N., Setälä H., Van Der Putten W.H., Wall D.H. (2004). Ecological Linkages Between Aboveground and Belowground Biota. Science.

[9] Wagg C., Bender S.F., Widmer F., Van Der Heijden M.G.A. (2014). Soil biodiversity and soil community composition determine ecosystem multifunctionality. Proc. Natl. Acad. Sci. USA.

[10] Wardle D.A., Bonner K.I., Barker G.M. (2002). Linkages between plant litter decomposition, litter quality, and vegetation responses to herbivores. Funct. Ecol..

[11] Adl M.S., Gupta V.S. (2006). Protists in soil ecology and forest nutrient cycling. Can. J. For. Res..

[12] Bennett J.A., Cahill J.F. (2016). Fungal effects on plant-plant interactions contribute to grassland plant abundances: Evidence from the field. J. Ecol..

[13] Hiiesalu I., Partel M., Dvison J., Gerhold P., Metsis M., Moora M. (2014). Species richness of arbuscular mycorhizal fungi: Associations with grassland plant richness and biomass. New Phytol..

[14] Pellissier L., Niculita-Hirzel H., Dubuis A., Pagni M., Guex N., Ndiribe C., Salamin N., Xenarios I., Goudet J., Sanders I.R. (2014). Soil fungal communities of grasslands are environmentally structured at a regional scale in the Alps. Mol. Ecol..

[15] Bahram M., Põlme S., Kõljalg U., Zarre S., Tedersoo L. (2011). Regional and local patterns of ectomycorrhizal fungal diversity and community structure along an altitudinal gradient in the Hyrcanian forests of northern Iran. New Phytol..

[16] Peay K.G., Baraloto C., Fine P.V.A. (2013). Strong coupling of plant and fungal community structure across western Amazonian rainforests. ISME J..

[17] Jumpponen A., Jones K.L. (2009). Massively parallel 454 sequencing indicates hyperdiverse fungal communities in temperateQuercus macrocarpaphyllosphere. New Phytol..

[18] Zheng C., Zhang S., Ragg S., Raftery D., Vitek O. (2011). Identification and quantification of metabolites in 1H NMR spectra by Bayesian model selection. J Bioinform. Adv..

[19] Nicholson J., Lindon J., Holmes E. (1999). “Metabolomics”: Understanding the metabolomics response of living systems to pathophysiological stimuli via multivariate statistical analysis of biological NMR spectroscopic data. Xenobiotica.

[20] Lu W., Clasquin M.F., Melamud E., Amador-Noguez D., Caudy A.A., Rabinowitz J.D. (2010). Metabolomic Analysis via Reversed-Phase Ion-Pairing Liquid Chromatography Coupled to a Stand Alone Orbitrap Mass Spectrometer. Anal. Chem..

[21] Figueira J., Gouveia-Figueira S., Öhman C., Holgerson P.L., Nording M.L., Öhman A. (2017). Metabolite quantification by NMR and LC-MS/MS reveals differences between unstimulated, stimulated, and pure parotid saliva. J. Pharm. Biomed. Anal..

[22] Ulrich E.L., Akutsu H., Doreleijiers J.F., Harano Y., Ioannidis Y.E., Lin J., Mading S., Maziuk D., Miller Z., Nakatani E. (2008). BioMagResBank. Nucleic Acids Res..

[23] Weljie A.M., Newton J., Mercier P., Carlson A.E., Slupsky C.M. (2006). Targeted Profiling: Quantitative Analysis of1H NMR Metabolomics Data. Anal. Chem..

[24] Khan M.S., Zaidi A., Ahemad M., Oves M., Wani P.A. (2010). Plant growth promotion by phosphate solubilizing fungi—Current perspective. Arch. Agron. Soil Sci..

[25] Britto D.T., Kronzucker H.J. (2002). NH4+ toxicity in higher plants: A critical review. J. Plant Physiol..

[26] Cedergren N., Madsen T.V. (2002). Nitrogen uptake by the floating macrophyte Lemma minor. J. New Phytol..

[27] Tylová E., Lorenzen B., Brix H., Votrubova O. (2005). The effects of NH_4_^+^ and NO_3_^−^ on growth, resource allocation and nitrogen uptake kinetics of Phragmites australis and Glyceria maxima. Aquat. Bot..

[28] Fang Y.Y., Babourina O., Rengel Z., Yang X.E., Pu P.M. (2007). Ammonium and nitrate uptake by the floating plant Landoltia punctate. Ann. Bot..

[29] Ashraf M., Foolad M. (2007). Roles of glycine betaine and proline in improving plant abiotic stress resistance. Environ. Exp. Bot..

[30] Matassini C., Parmeggiani C., Cardona F. (2020). New Frontiers on Human Safe Insecticides and Fungicides: An Opinion on Trehalase Inhibitors. Molecules.

[31] Elbein A.D. (1974). The metabolism of α,α-trehalose. Adv. Carbohyd. Chem. Biochem..

[32] Lunn J.E., Delorge I., Figueroa C.M., Van Dijck P., Stitt M. (2014). Trehalose metabolism in plants. Plant J..

[33] Crowe J.H. (2007). Trehalose as a “chemical champerone”. Fact and fantasy. Molecular Aspects of the Stress Response.

[34] Gancedo C., Flores C.-L. (2004). The importance of a functional trehalose biosynthetic pathway for the life of yeasts and fungi. FEMS Yeast Res..

[35] Valliyodan B., Nguyen H.T. (2006). Understanding regulatory networks and engineering for enhanced drought tolerance in plants. Curr. Opin. Plant Biol..

[36] Purvis J.E., Yomano L.P., Ingram L.O. (2005). Enhanced Trehalose Production Improves Growth of Escherichia coli under Osmotic Stress. Appl. Environ. Microbiol..

[37] Altamirano-Hernández J., Farías-Rodríguez R., Jaramillo V., Peña-Cabriales J. (2004). Seasonal variation in trehalose contents of roots and nodules of leguminous trees in a tropical deciduous forest in Mexico. Soil Biol. Biochem..

[38] Reina-Bueno M., Argandoña M., Nieto J.J., Garcia A.H., Iglesias-Guerra F., Delgado M.J., Vargas C. (2012). Role of trehalose in heat and desiccation tolerance in the soil bacterium Rhizobium etli. BMC Microbiol..

[39] Holmström K., Somersalo S., Mandal A., Palva E.T., Welin B. (2000). Improved tolerance to salinity and low temperature in transgenic tobacco producing glycine betaine. J. Exp. Bot..

[40] Ali Q., Ashraf M. (2011). Induction of Drought Tolerance in Maize (*Zea mays* L.) due to Exogenous Application of Trehalose: Growth, Photosynthesis, Water Relations and Oxidative Defence Mechanism. J. Agron. Crop. Sci..

[41] Holtmann G.J., Bremer E. (2004). Thermoprotection of Bacillus subtilis by Exogenously Provided Glycine Betaine and Structurally Related Compatible Solutes: Involvement of Opu Transporters. J. Bacteriol..

[42] Wishart D.S., Jewison T., Guo A.C., Wilson M., Knox C., Liu Y., Djoumbou J., Mandal R., Aziat F., Dong E. (2003). HMDB 3.0—The Human Metabolome Database. Nucleic Acids Res..

[43] Ichihashi Y., Date Y., Shino A., Shimizu T., Shibata A., Kumaishi K., Funahashi F., Wakayama K., Yamazaki K., Umezawa A. (2020). Multi-omics analysis on an agroecosystem reveals the significant role of organic nitrogen to increase agricultural crop yield. Proc. Natl. Acad. Sci. USA.

[44] Takeuchi Y., Ogasawara M., Kim S., Konnai M., Takematsu T., Suzuki A., Hyeon S., Che F., Furushima M. (1990). Promotive effect of Choline salts on groth of manila grass and bent grass. J. Jpn. Soc. Turf. Sci..

[45] Liu H., Cheng S., Logan B.E. (2005). Production of Electricity from Acetate or Butyrate Using a Single-Chamber Microbial Fuel Cell. Environ. Sci. Technol..

[46] Leroy F., De Vuyst L. (2004). Lactic acid bacteria as functional starter cultures for the food fermentation industry. Trends Food Sci. Technol..

[47] Yamanaka T. (2002). Corrosion by bacteria of concrete in sewerage systems and inhibitory effects of formates on their growth. Water Res..

[48] Inoki K., Zhu T., Guan K.-L. (2003). TSC2 NMediates cellular energy response to control cell growth and survival. J. Cell Sci..

[49] Kumari K., Singh R.P., Saxena S.K. (1987). Effect of Different Factors on the Movement of Some Amino Acids in Soils Using Thin-Layer Chromatography. J. Liq. Chromatogr..

[50] Zhang H., Jennings A., Barlow P.W., Forde B.G. (1999). Dual pathways for regulation of root branching by nitrate. Proc. Natl. Acad. Sci. USA.

[51] Masclaux-Daubresse C., Reisdorf-Cren M., Pageau K., Lelandais M., Grandjean O., Kronenberger J., Valadier M.-H., Feraud M., Jouglet T., Suzuki A. (2006). Glutamine sythetase-glutamate synthase pathway and glutamate dehydrogenase play distinct roles in the sink-source Nitrogen cycle in Tobacco. J. Plant Physiol..

[52] Amann R.I., Ludwig W., Schleifer K.H. (1995). Phylogenetic identification and in situ detection of individual microbial cells without cultivation. Microbiol. Rev..

[53] Torsvik V., Goksøyr J., Daae F.L. (1990). High diversity in DNA of soil bacteria. Appl. Environ. Microbiol..

[54] Nüsslein K., Tiedje J.M. (1999). Soil Bacterial Community Shift Correlated with Change from Forest to Pasture Vegetation in a Tropical Soil. Appl. Environ. Microbiol..

[55] Ward N.L., Challacombe J.F., Janssen P.H., Henrissat B., Coutinho P.M., Wu M., Xie G., Haft D.H., Sait M., Badger J. (2009). Three Genomes from the Phylum Acidobacteria Provide Insight into the Lifestyles of These Microorganisms in Soils. Appl. Environ. Microbiol..

[56] Borneman J., Triplett E.W. (1997). Molecular microbial diversity in soils from eastern Amazonia: Evidence for unusual microorganisms and microbial population shifts associated with deforestation. Appl. Environ. Microbiol..

[57] Visagie C.M., Seifert K.A., Houbraken J. (2014). Diversity of Penicillium section Citrina within the fynbos biome of South Africa, including a new species from a Protea repens infructescence. J Mycol..

[58] Frisvad J.C., Samson R.A. (2004). Polyphasic taxonomy of Penicillium subgenus Penicillium. A guide to identification of food and air-borne terverticillate Penicillia and their mycotoxins. Stud. Mycol..

[59] Pitt J.I., Hocking A.D. (2009). Fungi and Food Spoilage.

[60] Samson R.A., Yilmaz N., Houbraken J. (2011). Phylogeny and nomenculture of the genus Talaromyces and taxa accommodated in Penicillium subgenus Biverticillium. Stud. Mycol..

[61] Frisvad J.C., Smedsgaard J., Larsen T.O. (2004). Mycotoxins, drugs and other extrolites produced by species in Penicillium subgenus Penicillium. Stud. Mycol..

[62] Fleming A. (1980). On the Antibacterial Action of Cultures of a Penicillium, with Special Reference to Their Use in the Isolation of B. influenzae. Clin. Infect. Dis..

[63] Chain E., Florey H., Gardner A., Heatley N., Jennings M., Orr-Ewing J., Sanders A. (1940). Penicillin As a Chemotherapeutic Agent. Lancet.

[64] Abraham E., Chain E., Fletcher C., Gardner A., Heatley N., Jennings M., Florey H. (1941). Further Observations on Penicillin. Lancet.

[65] Thom C. (1945). Mycology present penicillin. J. Mycol..

[66] Van Der Heijden E.W., Kuyper T.W. (2003). Ecological strategies of ectomycorrhizal fungi of Salix repens: Root manipulation versus root replacement. Oikos.

[67] Baxter J.W., Dighton J. (2005). Phosphorus source alters host plant response to ectomycorrhizal diversity. Mycorrhiza.

[68] Broeckling C.D., Broz A.K., Bergelson J., Manter D.K., Vivanco J.M. (2007). Root Exudates Regulate Soil Fungal Community Composition and Diversity. Appl. Environ. Microbiol..

[69] Seastedt I.T., Callaway R.M., Pollock J.L., Kaur J. (2008). Allelopathy and plant invasions: Traditional, congeneric, and bio-geographical approaches. Biol. Invasions.

[70] Waqas M., Khan A., Kang S.-M., Kim Y., Lee S.-U. (2014). Phytohormone-producing fungal endophytes and hardwood-derived biochar interact to ameliorate heavy metal stress in soybeans. Biol. Fertil. Soils.

[71] Shivanna M.B., Meera M.S., Hyakumachi M. (1994). Sterile fungi from zoysiagrass rhizosphere as plant growth promoters in spring wheat. Can. J. Microbiol..

[72] Khan S.A., Hamayun M., Yoon H., Kim H.-Y., Suh S.-J., Hwang S.-K., Kim J.-M., Lee S.-U., Choo Y.-S., Yoon U.-H. (2008). Plant growth promotion and Penicillium citrinum. BMC Microbiol..

[73] Radhakrishnan R., Kang S.-M., Baek I.-Y., Lee S.-U. (2014). Characterization of plant growth-promoting traits of Penicillium species against the effects of high soil salinity and root disease. J. Plant Interact..

[74] Hossain M., Sultana F., Kubota M., Koyama H., Hyakumachi M. (2007). The Plant Growth-Promoting Fungus Penicillium simplicissimum GP17-2 Induces Resistance in Arabidopsis thaliana by Activation of Multiple Defense Signals. Plant Cell Physiol..

[75] Khan A.L., Hamayun M., Ahmad N., Hussain J., Kang S.-M., Kim Y.-H., Adnan M., Tang D.-S., Wagas M., Radhakrishnan R. (2011). Salinity Stress Resistance Offered by Endophytic Fungal Interaction Between Penicillium minioluteum LHL09 and Glycine max. J. Microbiol. Biotechnol..

[76] Swanepoel B., Bredenkamp G. (2006). The Vegetation Ecology of Ezemvelo Nature Reserve, Bronkhorstspruit, South Africa. Master’s Thesis.

[77] Kim H.K., Choi Y.H., Verpoorte R. (2010). NMR-based metabolomic analysis of plants. Nature.

[78] Maree J., Viljoen A. (2012). Phytochemical distinction between Pelargonium sidoides and Pelargonium reniforme—A quality control perspective. S. Afr. J. Bot..

[79] Mediani A., Abas F., Khatib A., Maulidiani M., Shaari K., Choi Y.H., Lajis N. (2012). 1H-NMR-based metabolomics approach to understanding the drying effects on the phytochemicals in Cosmos caudatus. Food Res. Int..

[80] Fernie A.R., Aharoni A., Wilmitzer L., Stutt M., Tohge T., Kopka J., Carol A.J., Saito K., Fraser P.D., Deluca V. (2011). Recommendations for reporting metabolite data. Plant Cell..

[81] Suzuki M., Nishiumi S., Kobayashi T., Azuma T., Yoshida M. (2016). LC–MS/MS-based metabolome analysis detected changes in the metabolic profiles of small and large intestinal adenomatous polyps in Apc Min/+ mice. Metabolomics.

[82] Matsubara A., Fukusaki E., Bamba T. (2010). Metabolite analysis by supercritical fluid chromatography. Bioanalysis.

[83] Xu W., Van Knegsel A., Saccenti E., Van Hoeij R., Kemp B., Vervoort J. (2020). Metabolomics of Milk Reflects a Negative Energy Balance in Cows. J. Proteome Res..

[84] Yang J., Schmelzer K., Georgi K., Hammock B.D. (2009). Quantitative Profiling Method for Oxylipin Metabolome by Liquid Chromatography Electrospray Ionization Tandem Mass Spectrometry. Anal. Chem..

